# Research on online public opinion dissemination and emergency countermeasures of food safety in universities—take the rat head and duck neck incident in China as an example

**DOI:** 10.3389/fpubh.2023.1346577

**Published:** 2024-02-01

**Authors:** Jinsi Liu, Su Wang, Zhihua Wang, Shixiang Chen

**Affiliations:** ^1^School of Politics and Public Administration, Wuhan University, Wuhan, China; ^2^Local Government Public Service Innovation Research Center, Wuhan University, Wuhan, China; ^3^College of Public Administration, South-Central Minzu University, Wuhan, China

**Keywords:** university food safety, rat head duck neck, life cycle theory, online public opinion, emergency measures

## Abstract

In recent years, food safety accidents have occurred frequently in colleges and universities, and students are prone to emotional resonance with food safety. It triggered heated discussions among the whole society and gradually formed a unique online public opinion on food safety in universities. After food safety incidents broke out in universities, some universities deliberately avoided responsibility or made mistakes in handling the incidents, which will create greater risks of online public opinion. Therefore, this paper takes the “Rat Head and Duck Neck” incident at Jiangxi Institute of Technology in China as an example. The purpose is to study the dissemination of public opinion on food safety online in universities and propose emergency countermeasures. Above all, the food safety online public opinion is divided into five stages: incubation period, burst period, spreading period, recurring period and dissipation period. Then, methods such as text mining and cluster analysis were used to deeply analyze the influencing factors at each stage of the development of food safety online public opinion. And analyze the role of different subjects in the development of public opinion based on the perspective of stakeholders. Finally, this paper provides corresponding countermeasures for different stages of online public opinion on food safety in universities, which provides suggestions and references for university governance. This study found that: (1) The resonance effect of online public opinion media on food safety in universities is significant. (2) Public opinion on food safety in universities is repetitive. (3) Improper response to food safety incidents in universities can easily trigger negative secondary public opinion.

## 1 Introduction

Currently, with the improvement of the economy and living standards, people pay more and more attention to healthy diets. In public health, foodborne diseases have become one of the threats to public health security. Physiological diseases caused by unhealthy food are explosive and aggregated, especially in developing countries, bringing a heavy disease burden (1). As an important educational institution in the country, colleges and universities are the foundation for building an educational power and are responsible for cultivating a group of intellectuals for society (2). Therefore, the healthy development of students has become a common concern of society. However, in recent years, food incidents in colleges and universities have occurred frequently, causing concerns among students and their families (3). As a gathering place for students, colleges and universities will pose a threat to the lives and safety of many students once a food safety incident occurs. At the same time, students begin to fear performance in the cafeteria, which also brings great psychological harm to students (4). In 2022, hundreds of Iranian students suffered from food poisoning, which damaged the credibility of universities and the government.^1^ There have also been many food safety incidents in universities in China. In September 2020, nearly a 100 students at Wuhu Vocational and Technical College in Anhui suffered from vomiting, diarrhea and other poisoning symptoms after eating in the canteen. This was caused by irregular food processing. Therefore, it can be seen that colleges and universities have become high-incidence areas for food safety accidents. For this reason, food safety in colleges and universities has also attracted much attention from the Chinese government. As early as 2003, China’s former Ministry of Health issued the “Emergency Notice on Strengthening the Supervision and Management of School Food Hygiene during SARS Prevention and Control” Given the dire food safety situation in colleges and universities, health and education administrative departments at all levels are required to conduct safety inspections in college canteens. Hygiene problems discovered during inspections should be corrected promptly to help schools improve their food hygiene management levels. In 2023, the State Council of China revised the “Measures for Evaluation and Assessment of Food Safety Work.” This aims to implement the “Four Strictest” requirements for food safety and strengthen local government territorial management responsibilities. Improve the ability to supervise the entire process from farmland to table, continuously improve the level of food safety throughout the chain, and ensure the health and life safety of the people.^2^ However, compared with food safety problems in other fields, food safety problems in universities have more serious consequences. The reason is that this poses a threat to the life and health of the majority of students, and its impact is sudden and widespread, which may further lead to the occurrence of mass infectious diseases. Food safety in colleges and universities is directly related to the lives and health of students, and it also involves the concerns of countless students’ families. At the same time, students, as the main active force on the Internet, will expose food safety accidents in school cafeterias and require schools to publicly solve food safety problems, thus forming a larger online public opinion on food safety in colleges and universities. After the outbreak of food safety accidents in universities, relevant departments intentionally avoided responsibilities or mishandled the incidents, thereby creating greater risks of public opinion on university food safety online.

Colleges and universities carry the important mission of “Educating Talents for the Country.” Sudden food safety incidents in universities endangering the health of students are negative news, which is completely different from the mission of “Educating Talents for the Country.” Negative news expresses stronger emotional energy on the Internet, directly contributing to the generation and development of online public opinion (5). Currently, with the advent of the 5G era, the Internet is gradually becoming more popular. According to the 52nd “Statistical Report on China’s Internet Development Status,” as of June 2023, the number of Internet users in China reached 1.079 billion, and the power of online public opinion is huge. After the outbreak of food safety incidents in colleges and universities, students, as the main force active on the Internet, actively discussed food safety incidents in colleges and universities. Students have a high degree of emotional resonance with emergencies in colleges and universities, so students will be exposed to food safety incidents in canteens around them on the Internet. Then the student union of our school forwarded and commented on the incident, which gradually attracted the attention and discussion of students from other schools and the whole society. People have condemned the school and asked officials to respond to the accident. As a result, the online public opinion on food safety in universities has been further fermented and developed. The outbreak of online public opinion on food safety in colleges and universities is sudden and spreading. This requires understanding the development rules of public opinion and responding in time. Otherwise, public opinion will develop in a bad direction and directly trigger mass incidents. At the same time, online public opinion on food safety brings harm to the reputation of universities and even threatens the normal teaching activities of schools. As the supervisor of universities, the government’s credibility will also be questioned. However, there are currently few studies on public opinion on food safety in universities. On June 1, 2023, the “Rat Head and Duck Neck” incident broke out at Jiangxi Institute of Technology in China. The incident triggered extensive discussions and generated a large public opinion on the university’s Internet. Therefore, based on the information life cycle theory and the perspective of stakeholders, this paper takes China’s “Rat Head and Duck Neck” incident as an example to try to explain the propagation rules of public opinion on food safety in colleges and universities, and propose appropriate emergency countermeasures for each stage of public opinion development. In this way, we can effectively prevent and control online public opinions on food safety in universities. The innovation points of this paper: (1) Creatively combines university online public opinion with food safety online public opinion. Take China’s “Rat Head and Duck Neck” incident as an example, which is very representative. It well explains the public opinion caused by food safety accidents in colleges and universities. (2) Using life cycle theory, the online public opinion on food safety in universities is divided into five stages. More importantly, text mining and cluster analysis methods are used to analyze the causes of online public opinion at different stages. On this basis, corresponding emergency countermeasures are given in a targeted manner.

## 2 Literature review

This paper focuses on the online public opinion on food safety in universities. However, there are few studies specifically studying online public opinion on food safety in universities. Compared with other types of online public opinions in universities, online public opinions on food safety in universities are unique, their scope of influence is wider, and their consequences are more serious. Therefore, this paper needs to study the evolution of public opinion on food safety in universities. With the further popularization of the Internet, it has become more convenient for college students to use social media to surf. Post comments on the Internet anytime and anywhere to discuss current hot events, thereby promoting the generation of hot searches on the Internet (6). Online public opinion in universities affects the image of universities, and the guidance and management of public opinions has become one of the daily tasks of universities. First of all, in the research on the evolution and dissemination of online public opinion in colleges and universities, college students have integrated into the wave of the Internet. College students actively discuss various social events on the Internet and are deeply affected by the online public opinion field (7). The generation of online public opinion in colleges and universities comes from hot events in colleges and universities, especially negative information about colleges and universities, which are most easily spread, thus triggering larger online public opinion (8). In the development of each online public opinion, the focus of netizens’ comments changes with the disclosure of information. It is very important to understand the themes of online public opinion at different stages, which has guiding significance for controlling the further expansion of public opinion (9). Therefore, using the Latent Dirichlet Allocation (LDA) topic model, based on the public opinion popularity index and time series-based trends, can perform a time series analysis of university online public opinions (6). Then, in order to resolve the crisis of online public opinion in universities, it is particularly important to explore the spread rules of online public opinion in universities. AnyLogic software was used to simulate the improved SNIDR infectious disease model, which can intuitively simulate the process of spreading public opinion on the university online (8). Of course, online public opinion in universities is often accompanied by rumors. In an environment with asymmetric information, rumors can easily spread. However, college students only pay attention to existing information on the Internet and comment on it, and do not know whether the incident is true and complete (10). The complex and ever-changing Internet environment challenges the moral concepts and values of college students. Especially for college students with immature physical and mental development, they are easily influenced by online public opinions. Among them, social motivations and information sources are the main factors affecting college students’ online public opinion dissemination behavior (11). Therefore, ideological and political education in colleges and universities should be combined with big data to guide college students’ outlook on life and values (12). In addition, colleges and universities should make full use of Internet tools to pay attention to student’s physical and mental health and behavior (13), so as to effectively use online public opinion to educate and guide them (14). Then, in the research on prediction and early warning of online public opinions in colleges and universities, it is mainly used to track and predict public opinions in a big data environment. Through the collection of university public opinion data, the public opinion information is then cleaned and processed. Then machine learning to track the theme of public opinion to achieve the detection of public opinion (15). College students are greatly affected by external public opinion and their self-awareness is not strong. Therefore, they need to increase their efforts to use big data to analyze the trend of public opinion to achieve early detection and early resolution (16). College students are independent individuals with high intellectual abilities. To demonstrate the spirit of individualism, it is very easy to express opinions and comments on hot social events, which has led to the emergence of online public opinions in colleges and universities (17). A multi-level and comprehensive dynamic detection system has been established using big data, which can effectively predict and process online public opinion in universities (18). Of course, the development of online public opinion in colleges and universities mainly includes three key entities: Internet media, college students, and management. Further analysis is the game between these three parties. The role of any party will affect the advancement of the entire public opinion (19). Among them, college students, as the core factor of online public opinion in colleges and universities, make inappropriate remarks on social platforms or become the disseminators of rumors. This directly leads to the generation and development of public opinion, for which it is necessary to establish a campus Weibo comment forwarding and comment prediction model based on neural networks (10). Schools pay attention to students’ interactive content on social media, and then understand students’ performance on online platforms, providing a basis for identifying and preventing potential problems (20). Finally, in the research on the processing and guidance of online public opinion in colleges and universities, for college students to develop healthily physically and mentally, it is very necessary to guide college students’ public opinions on social media, thereby promoting the formation of students’ correct values and outlook on life (21). Through semi-structured interviews with counselors, we can understand the factors that influence students’ participation in online public opinion, mainly subjective, objective and peripheral factors (22). In addition to paying attention to the online public opinion of universities themselves, it is more important to use big data to analyze the mental health of college students and establish an emergency protection system for the mental health of college students (23). For this reason, some scholars have been able to analyze the emotional attitudes of comments in public opinion based on the improved text emotion learning model, thereby controlling the emotional response of the entire public opinion event process (24). From the perspective of schools’ response to public opinion in colleges and universities, it is mainly divided into three stages: intervention beforehand, intervention during the incident and intervention after the incident. Topic guidance, rapid response and agenda-setting are more effective solutions (25). Universities can use smart campuses to establish public opinion platforms for monitoring and to analyze the generation and dissemination of public opinion (26). Of course, after online public opinion breaks out in universities, it will inevitably damage the image of universities, and repairing the image of universities is destined to be a long process (27). In particular, there are differences in the dissemination of online public opinion in different types of colleges and universities, and accordingly, targeted public opinion emergency countermeasures are needed.

So, this paper focuses on the online public opinion on food safety in universities. As the name suggests, it is the online public opinion caused by food safety accidents in university cafeterias. Compared with other types of online public opinions, online public opinions on food safety mainly involve conflicts between university cafeterias and student groups. As a group public place, university cafeterias have indoor air quality, environmental sanitation, food material sources and operating procedures that may cause problems in cafeteria hygiene (28). However, many studies only focus on questionnaire surveys on food safety in colleges and universities, and the subjects are students, canteen staff, and managers (29), and few directly study the online public opinions caused by food safety in colleges and universities. In addition, some scholars are currently studying online public opinion on food safety. In most cases, the media exposes relevant reports on food safety, which triggers heated discussions. For example, food fraud (30), green organic rice (31), alternative meat (32), genetically modified soybeans (33), etc. will arouse social concern. The underlying reason behind this is the consideration of safe food.

Of course, there is no doubt that similar to other online public opinions, the development of online public opinions in universities also proceeds in stages. Currently, it is more common to use life cycle theory to analyze the stage division of public opinion. The information life cycle is developed from life cycle theory (34). As early as the end of 1950, the crisis theory on the human life course was proposed, which was the prototype of the original life cycle theory (35). Currently, according to the life cycle theory, academic circles divide online public opinion into three, four (36), five (37) and six stages. Among them, the third and fourth stages (38) are the main ones. The difference is due to the different types of Internet public opinions. Internet public opinions triggered by different types of events have their own operating mechanisms, and their cycles are naturally also quite different (39). However, it is generally consistent with the life cycle theory, from creation to dissipation. Any period division is inseparable from three basic stages, namely: the formation period, development period, and decline period (40).

**Figure 1:**
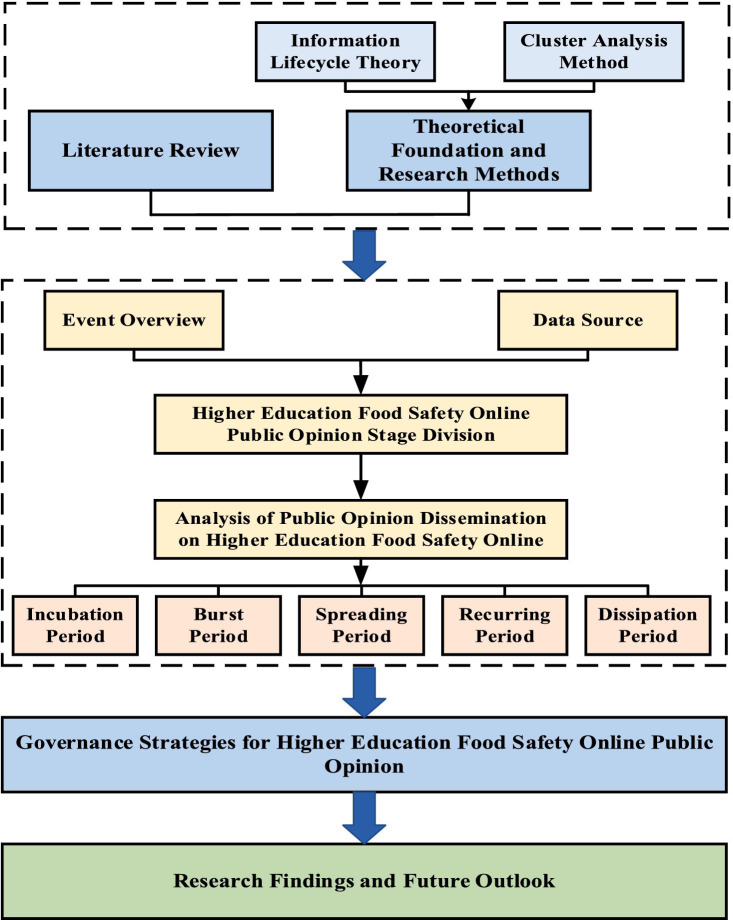
Research framework diagram.

Based on the above, there are currently many studies on online public opinion in colleges and universities. It mainly focuses on three aspects: the dissemination of university public opinion, the prediction and early warning of university public opinion, and the processing and guidance of university public opinion. However, it is regrettable that there are few research topics dedicated to online public opinion on food safety in universities. In addition, although there are a few studies on online public opinion on food safety, they are relatively broad and do not focus on schools as a public places. Therefore, this paper focuses on food online public opinion in universities. Apply life cycle theory and take the rat-headed duck-neck incident in China as an example. This paper provides a complete analysis of the development of public opinion on food safety accidents in colleges and universities. It aims to analyze the rules of the spread of public opinion on the Internet in colleges and universities and propose corresponding emergency countermeasures for each stage of public opinion. Before studying the online public opinion on food safety in universities, this paper puts forward the following assumptions. (1) The spread of online public opinions caused by food safety accidents in universities will not conflict with other types of online public opinions. (2) It is assumed that when discussing the role of each subject in public opinion, all factors considered have been comprehensive and other factors can be ignored. (3) This paper is based on Weibo data and ignores online public opinions on food safety in universities triggered by other platforms. The research framework of this paper (Figure 1).

## 3 Research methods and data sources

### 3.1 Cluster analysis method

This paper mainly uses text mining methods to analyze online public opinions on food safety in universities. Currently, cluster analysis is mostly used in text mining. Cluster analysis is a more advanced statistical method, and the most commonly used are hierarchical cluster analysis and K-means cluster analysis (41). The so-called cluster analysis is to divide the data set into several clusters according to the unsupervised principle, so that the subsets under the same cluster maintain a high degree of similarity (42). In text cluster analysis, the same cluster is a group containing similar topics, and objects in different clusters are as different as possible. This paper will use the FCM clustering algorithm. Compared with the hard ordinary C-means algorithm, the fuzzy C-means algorithm has been improved and is a soft clustering method. At the same time, this clustering method is clustering based on the objective function. In a given data set containing n data: $X=\left\{X_{1},X_{2},\cdots ,X_{i},\cdots X_{n}\right\}$. $X_{i}$ is the i-th feature vector. $X_{ij}$ is the j-th attribute of $X_{i}$. The FCM algorithm divides the data set into K categories, and the cluster centers are $\left[V_{1},V_{2},\cdots ,V_{n}\right]$. The objective function of FCM is formula (1), and its constraint is Formula (2).


(1) $$J\left(U,V\right)=\sum_{i=1}^{n}\sum_{j=1}^{k}u_{ij}^{m}d_{ij}^{2}$$

(2) $$\sum_{j=1}^{k}u_{ij}=1,u_{ij}\in \left[0,1\right]$$

In the above formula, $u_{ij}$ is the membership degree between the sample point $x_{i}$ and the cluster center $V_{n}$, m is the blur index. $d_{ij}$ is the distance between the sample point $x_{i}$ and the cluster center $V_{n}$.


(3) $$u_{ij}=\frac{1}{\sum_{c=1}^{k}{\left(\frac{d_{ij}}{d_{ik}}\right)}^{\frac{2}{m-1}}}$$

(4) $$v_{j}=\frac{\sum_{i=1}^{n}u_{ij}^{m}x_{i}}{\sum_{i=1}^{n}u_{ij}^{m}}$$

The fuzzy C-means algorithm obtains fuzzy classification of the sample set through iterative optimization of the objective function. To obtain the minimum value of the objective function J, the Lagrange multiplier method is used on the objective function under the condition that the constraints are met, and the membership matrix $u_{ij}$ and the cluster center $v_{j}$ are obtained (Formulas 3, 4).

### 3.2 Data sources

This paper focuses on the study of online public opinion on food safety in universities. The so-called online public opinion mainly refers to discussions on specific topics on major social platforms. Topic discussions eventually form larger online public opinions. Weibo is the most active platform on the Internet in China and has the widest audience. Therefore, this paper selects the Weibo platform as the data source for online public opinion on food safety in universities. Weibo topic popularity reflects the discussion and attention of Weibo users on a certain topic during a specific period. Generally, public opinion is intuitively evaluated from quantitative data such as the number of Weibo posts related to the topic, the number of forwards, the number of comments, and the number of likes (10). The number of retweets on Weibo is often used as a representation of the activity and popularity of Weibo. The higher the number of retweets, the higher the activity on Weibo (43). Therefore, in order to explore the evolution of online public opinion about the rat-headed duck-neck incident in Jiangxi colleges and universities in China, the keyword “Rat Head and Duck Neck” was entered on the Weibo page and Python software was used to crawl the data. Finally, relevant data such as the number of Weibo posts, topics, content, forwarding volume, comments, and likes were obtained. The “Rat Head and Duck Neck” incident occurred on June 1, 2023. After many turning points, public opinion stabilized and gradually subsided by the end of June 2023. Therefore, this study limited the time range of the data search to June 1, 2023, to June 30, 2023. After excluding irrelevant data, a total of 33,778 valid original Weibo posts, 138,717 valid retweets, 252,181 valid comments, and 4,037,052 valid likes were mined. The crawling time is July 1st, which can effectively ensure that the Weibo topic data is crawled, so that the Internet public opinion of the Rat Head and Duck Neck incident can be completely reproduced.

## 4 Empirical analysis of online public opinion dissemination of food safety incidents in universities

### 4.1 Event overview

**Figure 2:**
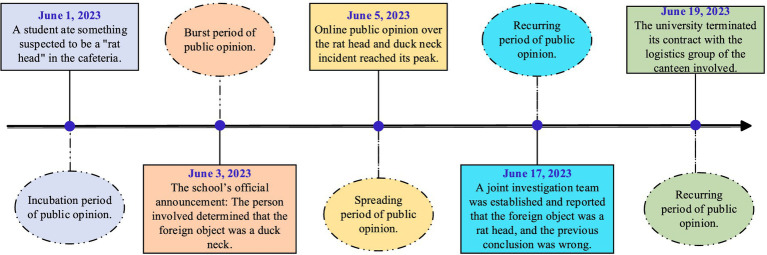
The development trajectory of the “Rat Head and Duck Neck” incident in universities in Jiangxi, China.

On June 1, 2023, a student at Jiangxi Institute of Technology in China was suspected of eating a foreign object in the cafeteria. Related videos were circulated on major platforms. The foreign object in the videos was highly similar to mice, which aroused social concern. The school and front-line law enforcement officers began to unanimously say that the foreign objects in the food were duck necks, which caused the Internet public opinion to reach a climax. Subsequently, a joint investigation team was established. After investigation and verification, the results showed that the conclusion given by the school was wrong. In the end, the school involved terminated its contract with the logistics group. After the entire incident was exposed that a student had eaten a foreign object, the university did not disclose the true situation truthfully, which made the public dissatisfied with the results of the report (Figure 2).

It can be seen from this that public opinion was not well handled in the initial stage, which made the online public opinion on food safety in colleges and universities reach a historical height. This public opinion lasted for a long time and aroused widespread concern from society. This has led the government to introduce a series of measures to control food safety in colleges and universities.

### 4.2 Stage division of online public opinion on food safety in universities

Different types of online public opinions have different evolution rules, and their peaks are also different. The same type of online public opinion will also be different due to the different progress of the event. With the continuous disclosure of information about the incident and the participation of different subjects of public opinion, public opinion will fluctuate. For example, some types of online public opinion are cyclical and will occur at every point in time. Some online public opinions are one-off and will only be generated when the event occurs. Of course, there are also Internet public opinions that will indirectly affect the frequent occurrence of other public opinions of the same type. This is the domino effect (44). Although the development trajectory of online public opinion is relatively complex, it still essentially conforms to the characteristics of the information life cycle. The generation of online public opinion basically comes from the outbreak of social or natural events, but the premise is that the event can attract public attention.

The “Rat Head and Duck Neck” incident in this paper belongs to the topic of food safety in colleges and universities, and food safety in colleges and universities is related to the health issues of students. The main subject of this incident was the university cafeteria, which has a certain degree of sensitivity. In addition, the development process of the incident itself was tortuous and complicated. The incident was questioned by students and then denied by the school and front-line law enforcement officials. Finally, the authoritative agency issued a document confirming that the school and front-line law enforcement officials made wrong judgments and other tortuous processes. The “Rat Head and Duck Neck” incident spans a long time, and it takes a certain amount of time for schools and relevant government agencies to investigate and collect evidence. This has caused public opinion on this topic to reach peaks repeatedly within a month, showing an evolutionary trend of “Multi-peak Public Opinion” However, it still essentially fits the cyclical and staged characteristics of the information life cycle. In order to carry out in-depth research, this paper is based on the information life cycle theory. Concerning the actual development of public opinion in the “Rat Head and Duck Neck” incident, the online public opinion of the “Rat Head and Duck Neck” incident is divided into five stages: the incubation period, the burst period, the spreading period, the recurring period, and the dissipation period. Specifically, the incubation period means that the incident has occurred and a certain amount of discussion has occurred, but it has not spread on a large scale. The burst period refers to the qualitative change or reversal of an event, or the event may be reported by the media, thereby attracting a large number of people to participate, and public opinion will spread rapidly in a short period, reaching the first peak of public opinion. The contagion period refers to that as information related to the incident continues to be disclosed, public opinion gradually deepens, and the number of subjects and content participating in the topic discussion continues to increase. This is manifested in the high growth rate of the number of Weibo posts, reposts, likes, and comments. The recurring period refers to the fact that after the first peak of public opinion appears, the public opinion does not completely subside, but peaks appear again as events evolve, or even multiple peaks appear. The dissipation period means that the issue of the public opinion incident is basically resolved and the official responds to the focus of public opinion. As time goes by, the discussion of the incident gradually decreases and the public opinion gradually dissipates.

**Figure 3:**
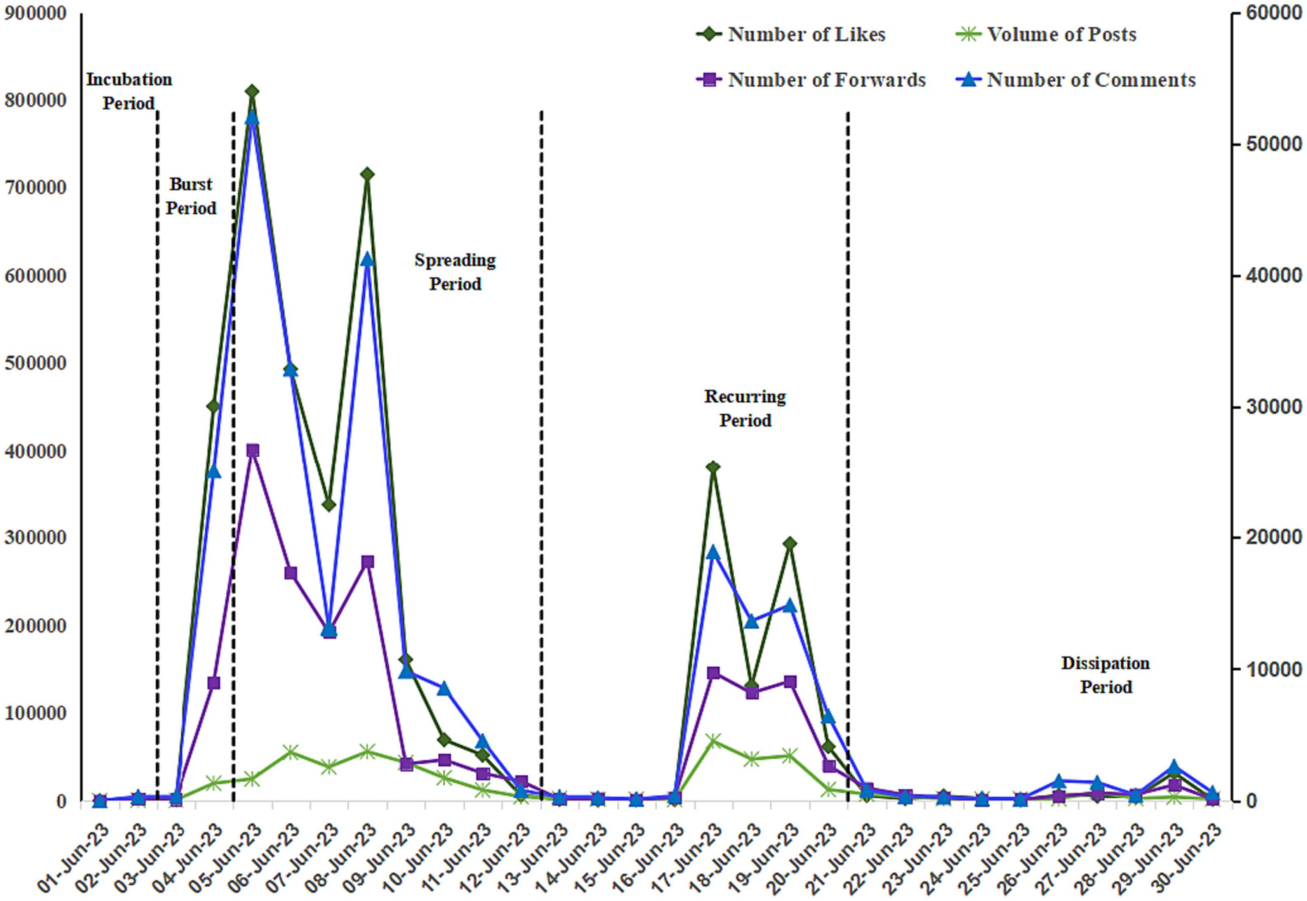
Stage division of online public opinion on the Rat Head and Duck Neck incident. (Number of Likes refers to the left coordinate axis, and the other 3 items refer to the right coordinate axis).

According to the number of Weibo posts, reposts, comments and likes of the “Rat Head and Duck Neck” incident, and also with reference to important events that occurred in the public opinion process, the Rat Head Duck Neck online public opinion was divided into five stages (Figure 3). Incubation period: June 1 to June 2, 2023, burst period: June 3 to June 4, 2023, spreading period: June 5 to June 12, 2023, recurring period: June 13, 2023–June 20, dissipation period: June 21, 2023–June 30. From the perspective of the number of posts, the number of likes, the number of comments, and the number of retweets, public opinion is mainly concentrated in three stages: the burst period, the spreading period, and the recurrence period, while the value of public opinion is lower in the incubation period and the dissipation period. On the whole, the incubation period of public opinion is short, and the outbreak period is characterized by a rapid increase in attention in a short period. The incident involves multiple stakeholders, causing public opinion to fluctuate in cycles. The dissipation period lasts longer than the outbreak period. In other words, the occurrence of online public opinion on food safety in universities is sudden, while its demise is often gradual.

### 4.3 Analysis of online public opinion dissemination of food safety in universities

The main body of online public opinion on food safety in universities is the issue of food safety in universities. At the same time, online public opinion in colleges and universities broke out concentratedly among the student groups. Therefore, the majority of public opinions in colleges and universities are related to online public opinions among students or between students and their schools (18). In the era of all media, the dissemination of online public opinion has broken through the limitations of time and space, and has the characteristics of fast propagation speed and wide diffusion range. The online public opinion triggered by the “Rat Head and Duck Neck” incident follows the information life cycle theory, and each stage presents relatively distinctive characteristics.

#### 4.3.1 Incubation period: public opinion spread in a small area contains risks

**Figure 4:**
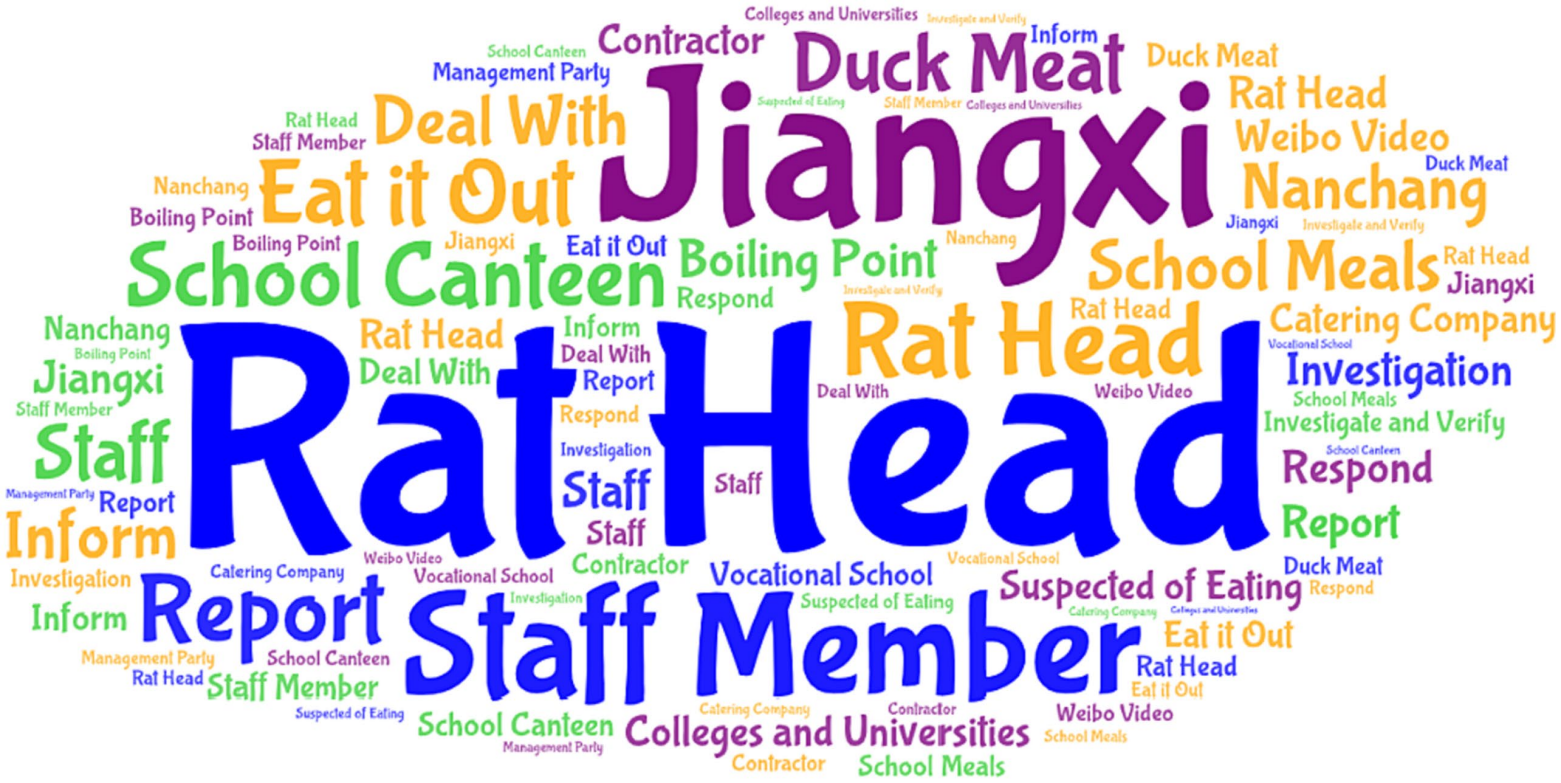
Word frequency chart of public opinion content during the incubation period.

The incubation period is the budding period of public opinion information. At this time, public opinion is only spread in a small range, with less media involvement, and has not yet received widespread attention from the public. The incubation period of the “Rat Head and Duck Neck” incident was short. The incident of a college student eating a rat-headed duck neck occurred on June 1. There was no relevant report on Weibo that day, and only some official media reported the incident the next day. On June 2, the total number of posts on Weibo was small, and the number of retweets, comments, and likes were all at a low level. Through the extraction and analysis of high-frequency words (Figure 4), the public opinion at this stage mainly has three aspects: Theme 1: #yue# disgusting#, the “Rat Head and Duck Neck” incident was initially spread in the form of video. Since the foreign object in the video is highly similar to a rat head, netizens expressed their discomfort with the foreign object after watching the video. This is the most intuitive and real feeling. Theme 2: #University#foodsafety#contractor# expresses netizens’ concerns about food safety in universities, especially their concerns that catering companies such as university contractors cannot guarantee food safety. The occurrence of food hygiene incidents in colleges and universities does not meet people’s expectations for colleges and universities, creating a good deficit before and after. Theme 3: #staff#investigationverification#, netizens hope to investigate and verify as soon as possible and report the truth of the incident. At the current stage, netizens view the incident more rationally and objectively, and the focus of the discussion is their concerns about the current status of food safety in universities. In other words, the practices and responses of universities at this stage have attracted much attention.

**Figure 5:**
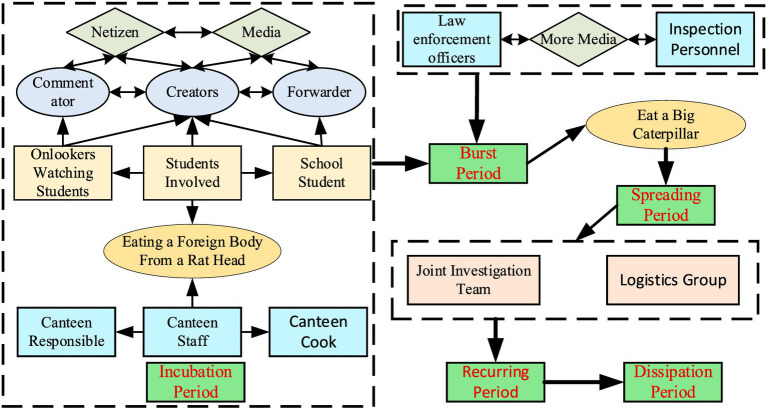
Diagram of stakeholder participation at various stages of public opinion.

The main reason why public opinion is relatively small at this stage is that the dissemination of information follows the life cycle theory, and the fermentation of public opinion requires a certain amount of dissemination time. The spiral of silence plays a significant role in the early stage of public opinion. The expression of personal opinions is a social psychological process. When people find that their opinions are consistent with those of the majority, they will tend to express their opinions. If your views do not conform to the attitudes of the majority, you will choose to remain silent. Especially when the event is not clear enough, even if they see the news about the event, more people tend to remain neutral and have a wait-and-see attitude. The “Rat Head and Duck Neck” incident has just occurred, and relevant reports are relatively limited. The full picture of the incident is still unclear, and the information is vague and sparse. Coupled with factors such as group pressure and herd mentality, people often choose to remain silent. From the perspective of relevant stakeholders (Figure 5), there are three main categories of stakeholders involved in the incubation period of public opinion. First of all, related information such as the suspected rat-head video will be disseminated by the students involved, students at the scene, and students of the school. Then, the school’s canteen workers, canteen directors, and canteen chefs represent the school and are the main actors directly related to the food safety incident. Finally, there are fewer media outlets with fewer headlines and fewer netizens. From the perspective of the three types of interest entities, it is obvious that the first two are in a state of game and there are conflicts between them. However, the media and netizens are in a wait-and-see attitude, looking at this incident with a casual attitude.

#### 4.3.2 Burst period: improper response triggers crisis of trust

The sudden period is the result of the accumulation of quantitative changes during the incubation period. At a certain point in time, the event reverses or changes, or the event is infinitely amplified by the media, causing the discussion of the topic to rise rapidly. At this time, the number of posts, reposts, comments and likes on the Weibo topic increased dramatically. On June 3, the university officially issued a post clarifying that the “Foreign Object” eaten by the students was a duck neck rather than a rat head. People responded to express doubts about this, which quickly set off a heated discussion among netizens. On June 4, the media conducted interviews and reports on the frontline law enforcement officers of the incident, and the law enforcement officers once again confirmed that the foreign object was a duck neck. This further intensifies the fermentation of public opinion and puts online public opinion at a higher risk. Through text mining at this stage, the representative discussion topics are: # Municipal Supervision Bureau once again confirmed that foreign matter in college meals is duck neck#, # Municipal Supervision Bureau responded to repeated comparisons and confirmed that it is duck neck#. The specific performance is as follows: the number of posts increased by 3247.5% compared with the previous day, the number of forwards increased by 8923.2%, the number of comments increased by 7140.2%, and the number of likes increased by 16099.9%. Public opinion at this stage was mainly questioning, targeting announcements issued by the school and feedback from frontline law enforcement personnel. The public expressed anger, doubt, and ridicule, conveying their distrust of government agencies, and negative public opinion heated up rapidly. At this stage, new high-frequency words in public opinion such as “Municipal Supervision Bureau,” “Credibility” and “Administration Bureau” appeared. The main focus of conflict has shifted from the food safety crisis to the crisis of trust in universities and the government. The attitude of netizens has shifted from worrying about food safety in universities to questioning the objectivity, fairness, and authority of government law enforcement personnel and law enforcement results.

**Figure 6:**
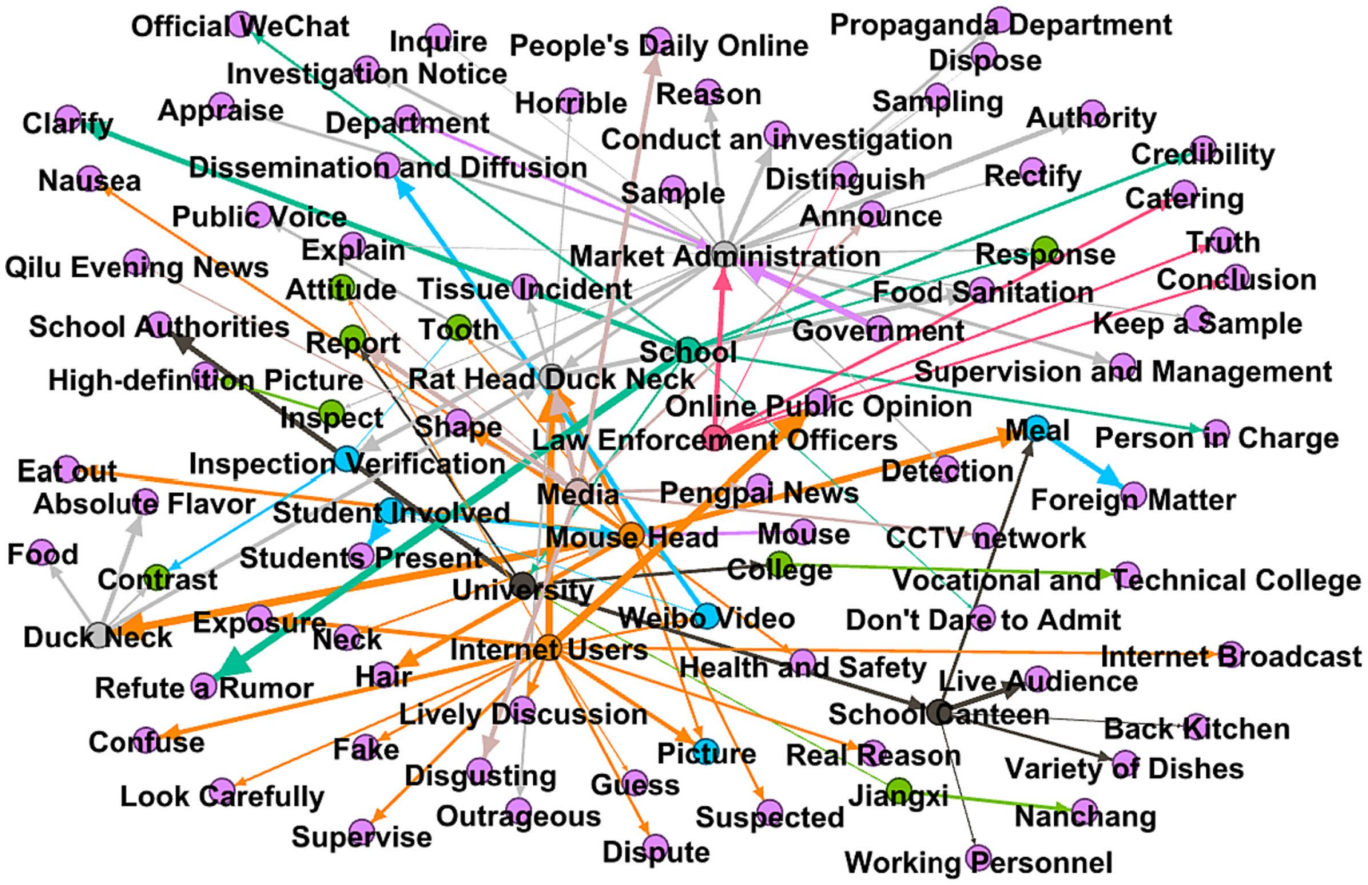
Clustering diagram of public opinion content during the burst period.

Cluster analysis of the content posted at this stage (Figure 6) is mainly based on different stakeholders. Rat head, duck neck, rat head, teeth, hair, shape, etc. are general descriptions of the video content. The university, school officials, school cafeteria, Jiangxi, Nanchang, etc. represent the school involved. Market Supervision Bureau, samples, in-depth investigations, etc. represent third-party testing agency departments. Netizens, media, Weibo videos, disgusting, etc. represent a large number of netizens and media participating in the discussion of public opinion. In addition, from the perspective of relevant stakeholders (Figure 5), compared with the public opinion incubation period, two new actors, law enforcement officers and testing agencies, have appeared in this stage, and they together form the fourth category of subjects. According to common sense, this type of subject has no interesting relationship with the first three types of subjects. Therefore, the canteen involved will be tested and investigated, and it should be trustworthy. However, the results announced by the test were surprising, and it was still confirmed that what was eaten was a duck neck rather than a rat head. This directly aroused public dissatisfaction, which in turn led to a rapid rise in public opinion. Of course, the media plays a role in fueling the situation. The media disseminates and analyzes the inspection results of law enforcement personnel and monitoring agencies, which creates a greater risk of online public opinion.

Analyzing the reasons for the explosive growth of public opinion at this stage. To begin with, the development of the Internet provides objective conditions for the rapid dissemination of public opinion. Compared with traditional media, new media platforms such as Weibo allow information to be disseminated faster and more widely. At the same time, the convenience of commenting and forwarding on Weibo can easily lead to the fission-type diffusion of online public opinion online, which is conducive to breaking the spiral of silence mechanism. Besides, the main body of public opinion dissemination is consistent with the main stakeholders of the incident. The main body of online public opinion dissemination is young students, and this college food safety incident first involves the safety of students. The two are similar in age, cultural level, living environment, interest views, etc., and are prone to emotional resonance. The last but not the least, improper response was the direct cause of the outbreak of online public opinion (45), and was also the direct trigger of the explosive growth of public opinion in the “Rat Head and Duck Neck” incident. In fact, after a university in Jiangxi issued a briefing on the situation on its official Weibo, the incident of “College Food Safety” has evolved into a secondary incident in which “School authorities and law enforcement officials are confusing right and wrong.” In this “Rat Head and Duck Neck” incident, the foreign object in the video is obviously a “Rat Head.” The school and front-line law enforcement officials all concluded that the foreign object was a duck’s neck, which made it difficult for the majority of netizens to believe it, and public opinion surged rapidly. Due to the secondary public opinion caused by improper government response, the public opinion caused by food safety management in universities originally belonged to the online public opinion in the social field. However, due to improper handling of the crisis by government departments, risks derived from public opinion are transmitted to the political field (46). This in turn causes netizens to question the fairness of law enforcement, and indirectly causes damage to the government’s credibility.

#### 4.3.3 Spreading period: the recurrence of similar canteen safety accidents extended the spreading period

The contagion period refers to the in-depth dissemination of information on social platforms (47). Both the subjects participating in public opinion and the number of topics discussed about the incident have reached unprecedented heights. Public opinion reached its peak on June 5, with the number of Weibo retweets, comments, and likes all reaching their highest peaks. In addition, on June 8, large caterpillars appeared in the dishes of the university cafeteria involved, which once again aroused public opinion and resonated with the original public opinion to form a peak of public opinion. The “Big Green Worm” incident broke the pace of public opinion’s original dissipation period and extended the duration of the contagion period. Online public opinion resonance refers to the convergence of some events due to their high similarity in the subjects involved, topics, and netizen sentiments. This then forms a cluster of public opinion events on the university food safety online, which are more likely to resonate with each other and produce a greater butterfly effect and resonance effect. There are connections and echoes between food safety incidents of the same type, and the occurrence of new food safety incidents can awaken earlier food safety incidents. The “Big Green Worm” incident resonated with the public opinion of the “Rat Head and Duck Neck” that has not yet ended, triggering a new climax of public opinion. The topic “A college student with a rat head and a duck neck claimed to have eaten a big caterpillar again” quickly set off a climax of public opinion. Compared with the peak of public opinion on June 5, the number of posts increased by 125.8%. In addition, the drop in the share price of Juewei Yabo has also become a hot topic in public opinion. Judging from the stakeholders participating in this event (Figure 5), compared with the burst period and the spreading period, there are no new increases in stakeholder groups (48). However, the rise in public opinion at this stage is mainly due to the large caterpillar eaten in the same canteen, which echoed the suspected rat heads eaten during the incubation period, so more media participated in related reports.

The attitudes of netizens can be divided into the following three types. The first is the rational group, which uses keywords such as “Investigation” and “Supervision” to express suggestions that the school should replace the canteen contractor as soon as possible. The group called on government departments to fulfill their regulatory obligations and conduct early investigations to give fair results. The second is the mocking group, which imitates the words and deeds of universities and law enforcement officials in the “Rat Head and Duck Neck” incident. Statements such as “After repeated comparisons, it is green peppers” and “Big caterpillars can enhance protein and provide students with extra meals” express their ridicule. The third group is the radical group, saying that the original video footage has left a deep impression on the majority of netizens. The written and oral statements provided by the Municipal Supervision Bureau cannot eliminate the strong impression caused by the original video. Therefore, the majority of netizens angrily questioned why universities and relevant departments regarded rats as ducks, why they did not strictly investigate the hygiene issues in university cafeterias, and how to ensure the food safety of university students. There are two main reasons for the formation of public opinion resonance. On the one hand, colleges and universities did not pay enough attention to this public opinion incident. While the “Rat Head and Duck Neck” public opinion has not dissipated, all food safety incidents in colleges and universities are considered sensitive matters at this time. At this stage, the colleges and universities involved did not pay enough attention to food safety issues, and failed to immediately investigate their own “Flammable and Explosive” points, which caused the “Big Green Worm” incident in the cafeteria to happen again. On the other hand, the elements of the two food safety accidents are highly similar. The incident in which big caterpillars were eaten again in a university is related to the same subject as the “Rat Head and Duck Neck” incident. They both belong to the field of public health in universities. Netizens’ sentiments are all negative. In other words, before the public opinion of the “Rat Head and Duck Neck” incident has subsided, the relevant subjects and similar incidents are in a highly sensitive period. Any similar emergencies can easily arouse the “Rat Head and Duck Neck “incident and form a resonance of public opinion.

#### 4.3.4 Recurring period: publicity of investigation results and in-depth media digging caused public opinion to fluctuate

The relapse period is the next stage after the spreading period of public opinion, where public opinion gradually declines after a long peak. Because the food safety incident had not been completely resolved, public opinion remained silent for a relatively short period. However, as more new details and content are disclosed, or as the incident progresses, public opinion will reach a climax again. With the follow-up reports of the “Rat Head and Duck Neck” incident, the media dug into the relevant information of the cooperative canteen, which aroused heated discussions among netizens and brought about two sub-peaks of public opinion (Table 1).

**Table 1:** Introduction to the peak information of public opinion recurrence period.

Peak	Time	Key Events	Volume of Post	Difference	Forward volume	Difference	Volume of comments	Difference	Volume of likes	Difference
Crest One	17/6	Joint investigation team announces results	4,530	+172.1%	9,873	−63.4%	19,247	−63.2%	381,711	−53.0%
Crest Two	19/6	Logistics Group operates more than 700 university canteen projects	3,426	+105.8%	9,102	−66.3%	14,872	−71.6%	292,954	−63.9%

On June 17, the joint investigation team reported the investigation status and confirmed that the conclusion that the foreign object was a duck neck was wrong. In other words, this refutes the investigation conclusions previously announced by the university and front-line law enforcement officials. At the same time, a large number of media spread this reversal of the findings. The inconsistencies between the two surveys were highlighted. This aroused public suspicion and dissatisfaction with the preliminary investigation. Public opinion quickly rebounded, forming the first peak in the public opinion cycle. Compared with the peak of public opinion on June 5, the number of posts increased by 172.1%, while the number of forwards, comments, and likes decreased by 63.4, 63.2, and 53%, respectively. The announcement of this investigation means the end of the “Rat Head and Duck Neck” incident, and the outcome of the incident can be reversed. The previous investigations by law enforcement officials and universities were wrong, and the credibility of the Nanchang Municipal Supervision Bureau and the universities involved was once again damaged. At the same time, the joint investigation team was able to respond positively to avoid further deterioration of public opinion and trigger the public to think rationally about the incident. Representative posts: #People’s Daily Online: The more authentic, the more authoritative, the inspiration from the “Rat Head and Duck Neck” incident#. Ange Rui’s comment on the #rat-headed-duck-neck incident forwarded by China Changan Network warned that we must adhere to seek truth from facts#, which accelerated public opinion into a period of dissipation.

**Figure 7:**
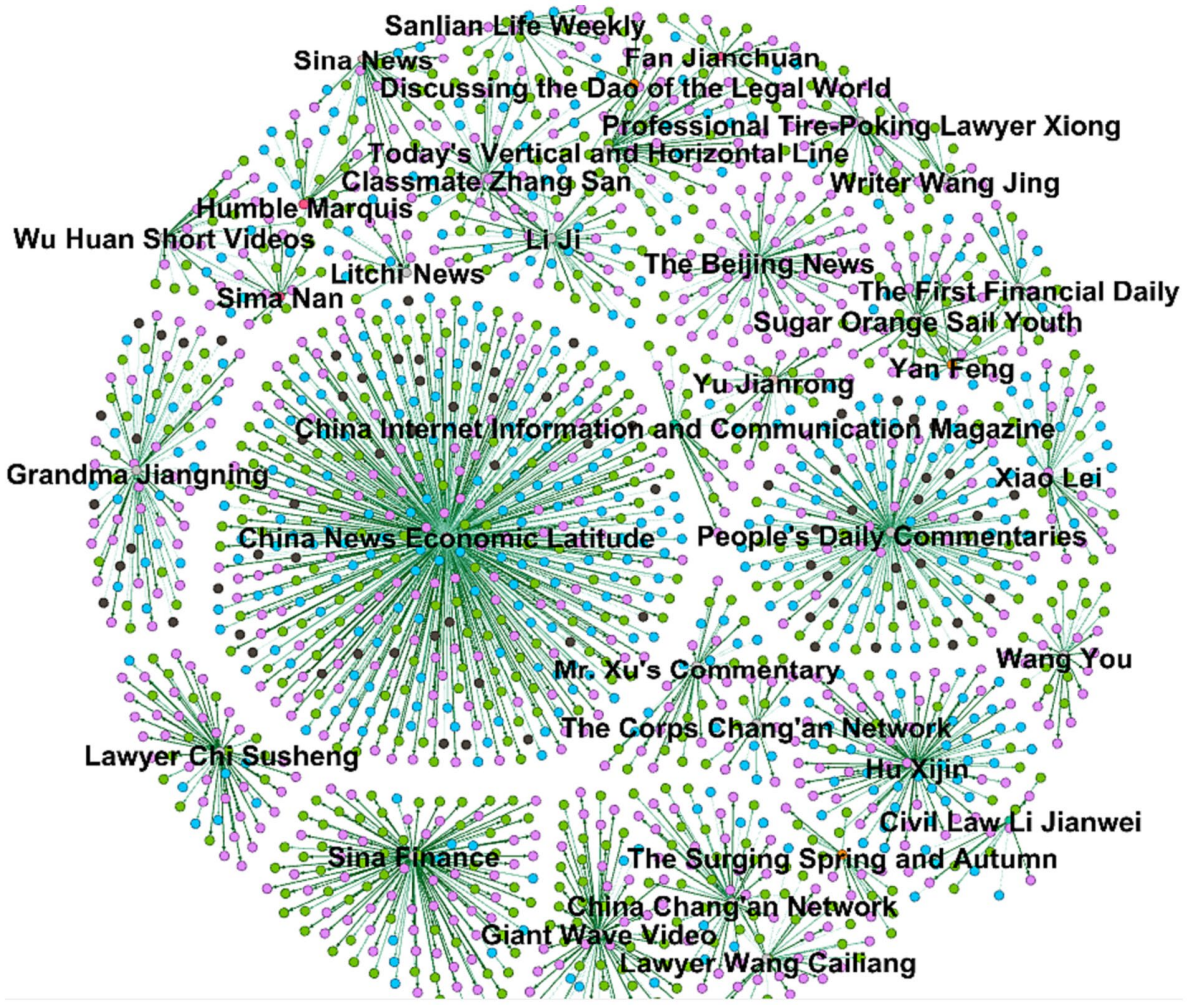
Media post forwarding path diagram.

Because both universities and grassroots law enforcement officials believe that foreign objects are ducknecks, netizens question the collusion of interests between the school, the government, and the logistics group. As a result, the media have dug out relevant information about the logistics group involved in the university cooperation and reported on it (Figure 7). It can be seen from this that the media and self-media play a greater role at this stage. Through the continuous disclosure of information related to the rat head and duck neck incident, it has been forwarded by the public, resulting in two sub-peaks of public opinion during the cycle of public opinion. According to relevant media reports, the logistics group operates more than 700 college canteens, which is in sharp contrast to the “Rat Head and Duck Neck” incident that occurred this time. It once again set off waves of public opinion and became the second peak of public opinion in the period of repeated public opinion. Compared with the peak of public opinion on June 5, the number of posts increased by 105.8%, while the number of forwards, comments, and likes decreased by 66.3, 71.6, and 63.9%, respectively. Of course, compared to Peak 1, the number of retweets, comments, and likes have all declined. This shows that the government’s positive response to expose the truth is conducive to controlling public opinion, while the distortion of facts becomes an accelerator for the growth of public opinion. More importantly, public opinion fluctuates repeatedly. Analyzing the reasons, on the one hand, as events develop or change, new information is constantly being disclosed. On the other hand, in the virtual online world, a large amount of false information or rumors will spread (49). Of course, social bots also exist in social platforms. These social robots are used by malicious people to publish misleading and misleading information. These are not information or rumors, but they will be forwarded by a large number of self-media and netizens, thereby expanding the spread of rumors (50). For example, in this incident, many netizens suspected that there was a transaction between the school and the Houqing Group. As it spread, this seemed to directly confirm the news, but there was no actual evidence (10). However, the generation and spread of rumors directly promote the increase in the risk of online public opinion (51).

The main driving force behind this peak of public opinion is the media’s agenda-setting and the complication of stakeholder relations. On the one hand, the media reports by digging into more information about the subjects related to the incident. After the rat-headed duck-neck incident came to a final conclusion, netizens could not help but wonder what force caused many parties to refer to the rat as a duck. China Kuai Group was the canteen involved, and it was one of the subjects that netizens focused on. After the Rat Head and Duck Neck incident came to an end, to continue to obtain corresponding traffic, the media dug out more information about the group in an attempt to attract public attention. The media set a Weibo topic: #founder once said that Zhongfa Catering is a leading brand in the canteen industry#. The companies involved in # “Rat Head and Duck Neck” are exposed! The parent company operates more than 700 college canteens across the country#. Keywords such as “Leading Canteen Brand” are in sharp contrast to the “Rat Head and Duck Neck” incident. Data from more than 700 college canteens once again aroused discussions among netizens. On the other hand, universities play a special and important role in food safety. They are both supervisors of canteen contractors and are also supervised by relevant government departments.

#### 4.3.5 Dissipation period: the declining public opinion leaves more reflections

During the dissipation period, no new tipping point emerged, and public opinion gradually became more rational, mainly focusing on two aspects. Topic 1: #Canteen#Involved#Enterprise#Parent Company#. The company still focuses on catering companies that cooperate with the universities involved. Because the company cooperates with many universities, and front-line law enforcement officials from the school and the government initially said that the “Foreign Object” in the canteen was a duck neck, this caused netizens to question the collusion between government and business. Topic 2: #University#foodsafety#ring the alarm#. Netizens and related media are worried and thinking about how food safety in universities should be ensured. They call that this rat-head-duck-neck incident should also sound the alarm for other universities.

In the dissipation period, the popularity of online public opinion caused by the rat-headed duck-neck gradually weakened, and its essence was that the public opinion incident was resolved. The dissipation period of public opinion also indicates the end of the recurrence period, and the sub-peaks that appeared in the recurrence period are all declining. This indicates that there will be no new information disclosure, and thus no new peak of public opinion. Public opinion in the dissipation period has always existed but at a relatively low level. At this stage, netizens’ attention to topical events continues to decrease, and public opinion gradually subsides and enters a controllable stage. Analyzed from another perspective, as the joint investigation team released the real announcement, Jiangxi Industrial Vocational and Technical College has terminated its contract with Jiangxi Zhongkuai Logistics Service Co., Ltd. This shows that the public participated in this public opinion victory and public opinion promoted the democratization process. The rat-headed duck-neck incident is not only related to the company that contracted the canteen but also is inseparable from the school’s canteen management model. More importantly, it is even more puzzling that the school and grassroots law enforcement teams did not disclose the truth at first, but instead covered up the facts. However, during the period when public opinion dissipated, the government, schools and other officials did not punish the relevant responsible persons involved, and did not fundamentally change the problems existing in the rat-headed duck-neck incident. Therefore, this highlights the necessity of this study. On the one hand, how should the government and school officials better supervise the hygiene of school cafeterias? On the other hand, in the face of public opinion in colleges and universities, the government and school officials should respond to public opinion as soon as possible. And solve it in time to prevent public opinion from reaching an uncontrollable point.

## 5 Strategies for managing online public opinion on food safety in universities

Through the analysis of the various stages of public opinion dissemination, it is found that the stakeholders participating in public opinion, topic discussions and public opinion risks are different at different stages, so the governance strategies cannot be generalized. Specifically, the incubation period should identify risks and actively intervene in incident investigations. During emergencies, we should face problems head-on and pursue fairness and objectivity. During the spreading period, we should be highly vigilant and proactively investigate potential risks. During the recurrence period, follow-up should be carried out promptly and positive responses should be given to dismissal. During the dissipation period, the supervision system should be improved to solve the problem from the root cause (52).

### 5.1 Incubation period: identify risks and actively intervene in incident investigation

During the incubation period, when public opinion has not spread on a large scale, it is the golden period to control the dissemination of public opinion. The following two points should be achieved at this stage: In the first place, identify risks. Colleges and universities are gathering places for intellectuals, and students have a special identity. They are not only college students with a greater awareness of rights protection, but also Internet users who actively participate in social platforms. Therefore, online public opinion in universities has a wide range of influence and lasts for a long time, and it is easy to trigger large-scale public opinion. Food safety is related to health and is also a topic of great concern to the public. Therefore, colleges and universities should conduct food safety inspections, regularly inspect the operation of canteens, increase penalties for canteens, and provide students with more channels to safeguard their rights (53). Once a food safety incident is discovered, identify risks as early as possible and adopt multiple contingency plans to intervene as soon as possible (54). The second is timely intervention and correct response. The parties involved in food safety incidents in universities include universities, canteens that cooperate with universities, and the government. Universities are the primary supervisory bodies of their cooperative canteens. They should proactively intervene in the incident and investigate the canteens involved, and publish the results of the investigation promptly, and release corresponding details when necessary. As the supervisor of food safety in colleges and universities, the government should also intervene promptly to conduct investigations, handle the incidents fairly and objectively, and fulfill its supervision obligations (55).

### 5.2 Burst period: face problems head-on and pursue fairness and objectivity

The outbreak period of public opinion is the stage when netizens express their emotions. Facing the emotions and demands of netizens directly is the best way to stabilize public opinion. Firstly, establish a public opinion emergency management system. When public opinion enters an outbreak period, corresponding public opinion data must be collected and sent to the expert group, which analyzes the existing data and past cases in the case library. Analyze the categories and development trends of public opinion, provide public opinion emergency plans and detail work arrangements (56). Secondly, respond positively to the issues that netizens are concerned about. Analyze and clarify the concerns and conflicts of netizens, grasp the key to the problem and respond to it (57). Taking this “Rat Head and Duck Neck” incident as an example, netizens unanimously believed that the foreign object in the video was a rat head, and the school and the government should provide true investigation results. Thirdly, the announcement of investigation results adopts a leader-responsibility system. To avoid further development of the situation or the occurrence of secondary incidents, formal incident investigation results need to be released promptly. And adopt a leader responsibility system, the implementation of which is conducive to improving the fairness of investigations. Finally, attention should be paid to the guidance of public opinion by opinion leaders. Authoritative comments have a guiding role, calling on mainstream media and authoritative self-media to correctly guide online public opinion and guide netizens to be objective and rational.

### 5.3 Spreading period: be highly vigilant and proactively investigate potential risks

During the spreading stage, netizen attention reaches its peak, hot topics continue to deepen, and more information continues to be disclosed. At this stage, you should be highly vigilant about the situation turning to the worse side (24). Therefore, three things need to be done at this stage. Above all, in the relatively complex period of public opinion spread, we must be able to grasp the focus of the public opinion debate at this stage and be able to prepare solutions to the focus issues. Then, we need to return to the investigation of the rat head and duck neck incident, strive to draw conclusions as soon as possible and publish a response, and take practical actions to answer the public’s doubts. At last, we must pay attention to the health and safety of existing canteens to avoid similar incidents from happening again. The university where the incident occurred and the cooperating logistics group should promptly investigate potential food hygiene hazards. Other universities and logistics groups should also sound the alarm and proactively investigate and improve relevant food safety supervision systems (53).

### 5.4 Recurring period: timely follow-up, positive response to dispel doubts

The tortuous development of the incident and the occurrence of secondary incidents will lead to a period of repeated public opinion. In food safety incidents in universities, inappropriate responses from the government or universities will break the pattern of public opinion entering a subsidence period. The rising voices of doubts from netizens have pushed public opinion into a period of recurrence. The essence of the existence of the period of recurrence is that the problem of the incident has not yet been completely resolved. Although the joint investigation team announced relatively realistic results, the public is still confused about the previous school authorities and grassroots law enforcement officers. Therefore, this stage needs to focus on resolving secondary incidents that cause fluctuations in public opinion, responding to relevant issues in a targeted manner, and taking measures to punish the subjects involved when necessary (25). Of course, the subsequent progress of the incident should be disclosed promptly at this stage. Publicizing the details and progress of the handling of the incident by holding media meetings and other forms, and responding positively to netizens’ questions will help public opinion enter the fading period as soon as possible (21).

### 5.5 Dissipation period: improve the supervision system and solve the problem from the root cause

During the subsidence period of public opinion, the development of things has come to an end, and the situation has been controlled to a certain extent. In the absence of new stimulus points, new public opinion crises will not arise again. At this stage, attention should be paid to the continuous tracking of evaluation feedback of public opinion events and tracing the source (23). The first is to establish an evaluation and feedback mechanism for public opinion, analyze the characteristics of each life cycle stage of public opinion, and sort out the causes of public opinion crises. Analyze the key nodes of public opinion control, evaluate and reflect on whether the responses of each subject are appropriate, and think about how to respond to similar emergencies in the future (58). The second is to continue to track public opinions and be alert to the occurrence of new public opinions. Continue to track sensitive words that can easily trigger public opinion crises, and be careful not to enter a period of repeated public opinion. The third is to get to the root of the problem. The frequent occurrence of food safety problems in colleges and universities has exposed the current backwardness of food safety supervision, imperfect supervision systems, and even whether the standards and procedures for the introduction of canteens are scientific. Corresponding supervision systems, canteen introduction processes and standards should be improved. Solve the problem fundamentally and avoid the occurrence of public opinion arousal effect and resonance effect (53).

## 6 Research conclusions and future prospects

### 6.1 Research conclusions

Through the research of this paper, the following conclusions can be drawn: (1) The Development of Online Public Opinion on Food Safety in Universities is in Line With the Life Cycle Theory. This paper uses the life cycle theory to explain the entire process of the development of Shutou Yabo online public opinion. According to this theory, online public opinion is divided into five stages. The causes and characteristics of online public opinion in different stages are different. At the same time, the use of text mining, especially the use of cluster analysis methods, can effectively analyze the content of public opinion at various stages of public opinion. On this basis, proposing corresponding emergency countermeasures for each stage of online public opinion on food safety in universities can achieve very good results. This provides a new idea for the government or school officials to prevent and control online public opinion in colleges and universities. (2) The Resonance Effect of Online Public Opinion Media on Food Safety in Universities is Significant. The “Resonance Effect” refers to the fact that mainstream media have a large number of users and fans, etc., and act as public opinion leaders when reporting official news, affecting the discourse direction of other media. During the development of this “Rat Head and Duck Neck” incident, the agenda-setting among the media also referred to each other’s reporting content. The agenda-setting always flowed from the more authoritative media to other media. Mainstream media such as People’s Daily Online and Toutiao News have established a dominant position in information dissemination with their strong influence. After the mainstream media publishes the report, it triggers other media to forward or follow-up reports based on the discourse. Similar information is reposted in large numbers in a short period. In the Weibo reports reported by mainstream media, there is a high degree of consistency in reporting time, labels, genres, forms, attitude tendencies, etc., creating a resonance effect between media. (3) The Development of Online Public Opinion on Food Safety in Universities is Repetitive. There were a total of four public opinion peaks in the “Rat Head and Duck Neck” incident, that is, there were periodic recurrences of public opinion. The first spike in public opinion occurred because universities and the government responded inappropriately. The second peak of public opinion occurred because similar incidents occurred again, and the recurrence of similar incidents can easily awaken earlier food safety incidents and form a resonance of public opinion. The third peak of public opinion occurred because the joint investigation team announced its investigation results and the incident itself took a turn. The reason for the fourth peak of public opinion was that the media dug into relevant information about the college cafeteria involved. Analysis revealed that there are various reasons for the recurrence of public opinion on Weibo. In addition, the first peak of public opinion is the highest, and the subsequent three peaks of public opinion decrease in sequence. (4) A Fair Response is Conducive to Promoting the Dissipation Period of Negative Public Opinion in Universities. In public opinion on food safety incidents, inappropriate responses from relevant subjects may directly push public opinion to a peak, triggering the rapid growth and spread of negative public opinion. In the “Rat Head and Duck Neck” incident, the school and the government ignored the facts and responded inappropriately, triggering the rapid spread of negative emotions among netizens. Moreover, the original focus of public opinion on food safety has shifted to the collusion of interests between the school and the government, which has reduced the government’s credibility. When the joint investigation team announced the truth of the incident, it pushed public opinion into a dissipation period. Therefore, responding fairly and facing the doubts of netizens directly will help push negative public opinion into a dissipation period as soon as possible.

### 6.2 Future prospects

This study has certain shortcomings, and the data sources studied in this paper are limited. We selected data from the Weibo platform about the “Rat Head and Duck Neck” incident for research. However, the Weibo platform is only one of many social platforms, and it is difficult to fully reflect all public opinions on the rat head and duck neck incident. There may be certain deviations between the summary of online public opinion patterns regarding this incident and the actual situation. In future research, the discussion on the resonance effect of public opinions on multiple food safety online can be strengthened. This includes the mechanisms and scope of impact between food safety incidents. In addition, after the outbreak of food safety emergencies in universities, public opinion may have experienced multiple cycles due to the complexity of society and the tortuous nature of the incident itself. Therefore, the multi-cycle pattern of online public opinion on food safety is also worthy of further exploration. For example, compare the reasons for the emergence, duration, and scope of influence of multi-cycle public opinions.

## Data availability statement

The datasets presented in this study can be found in online repositories. The names of the repository/repositories and accession number(s) can be found in the article/supplementary material. The data is also available at https://doi.org/10.6084/m9.figshare.24638799.v2.

## Author contributions

JL and SW: Conceptualization, Methodology and Formal Analysis. JL and ZW: Investigation, Original draft Preparation and Review and Editing. SC: Supervision and Funding acquisition. All authors contributed to this work and approved the submitted version.

## Footnotes

^1^Data Sources: https://www.universityworldnews.com/.

^2^Data Sources: https://www.pkulaw.com/law.
